# A privacy-protecting eggplant disease detection framework based on the YOLOv11n-12D model

**DOI:** 10.3389/fpls.2025.1634408

**Published:** 2025-10-10

**Authors:** Jiao Han, Zhenzhen Wu, Yandong Ding, Yantong Guo, Rui Fu

**Affiliations:** Weifang University of Science and Technology, Weifang, China

**Keywords:** image encryption, eggplant disease detection, YOLOv11n-12D, privacy protection, intelligent agriculture

## Abstract

The growing global population and rising concerns about food security highlight the critical need for intelligent agriculture. Among various technologies, plant disease detection is vital but faces challenges in balancing data privacy and model accuracy. To address this, we propose a novel privacy-preserving eggplant disease detection system with high accuracy. First, we introduce a lightweight 3D chaotic cube-based image encryption method that ensures security with low computational cost. Second, a streamlined YOLOv11n-12D framework is employed to optimize detection performance on resource-constrained devices. Finally, the encryption and detection modules are integrated into a real-time, secure, and accurate identification system.Experimental results show our framework achieves near-ideal encryption security (entropy=7.6195, Number of Pixel Change Rate(NPCR)=99.63%, Unified Average Changing Intensity(UACI)=32.85%) with 23× faster encryption (0.0127s) versus existing methods. The distilled YOLOv11n-12D model maintains teacher-level accuracy (mAP@0.5=0.849) at 3.6× the speed of YOLOv12s (2.7ms/inference), with +6.5% mAP improvement for small disease detection (e.g., thrips). This system balances privacy and real-time performance for smart agriculture applications.

## 1 Introduction

With the rapid advancement of agricultural digitalization, crop disease detection has become critical for ensuring food security and improving agricultural productivity (Elijah et al., 2018; Cornia, 1985). In many remote or underdeveloped regions, due to the lack of professional expertise and detection equipment, farmers often transmit crop images to external agricultural centers for manual or automated analysis (Baldi and La Porta, 2020). However, existing methods frequently struggle to balance model accuracy with data privacy protection. In addition, the detection systems must operate efficiently on resource-constrained devices typical of rural environments, while ensuring secure handling of sensitive data during transmission and storage. Consequently, the development of efficient, automated, and privacy-preserving disease detection systems that are both lightweight and reliable is crucial for promoting smart agriculture.

In recent years, deep learning-based object detection algorithms have achieved remarkable progress in plant disease recognition (Senthil Pandi et al., 2024; Ravì et al., 2017; Zhang et al., 2017). Lightweight models, particularly the YOLO series, have attracted considerable attention for their fast detection speeds and high accuracy (Zhang et al., 2024; He et al., 2024; Liu et al., 2022). Sangaiah et al. (2024) proposed T-YOLO-Rice, based on YOLOv4, to improve small-target detection such as rice leaf spots, outperforming YOLOv7 but remaining limited to a single task. To address diverse diseases and class imbalance, Roy and Bhaduri (2022) developed Dense-YOLOv4 by integrating DenseNet and an enhanced PANet, achieving 96.20% mAP and 93.61% F1-score for mango disease detection, and demonstrating generalization to grape and tomato diseases. Lin et al. (2023) built YOLO-Tobacco based on YOLOX-Tiny by adding HMU and CBAM modules, improving outdoor tobacco leaf detection (80.56% AP, 69 FPS), although its adaptability to multiple diseases remains limited. Building upon these advances, Li et al. (2023a) introduced MG-YOLO, integrating multi-head self-attention, BiFPN, and GhostCSP modules, achieving 98.3% accuracy at 0.009 seconds per image and surpassing YOLOv5 by 6.8% in complex environments. In addition to single-task detection, recent studies have explored joint detection and tracking paradigms (Li et al., 2023b, 2024), leveraging reinforcement learning to achieve object recognition and continuous tracking in dynamic environments. For example, Li et al. (2025) proposed a reinforcement learning-based joint detection and tracking paradigm for compact HFSWR target detection and tracking, which effectively improves detection probability and tracking performance.

In image tasks related to object detection, image enhancement has also emerged as an important research direction in recent years. For example, researchers have proposed a reinforcement learning-based human visual perception-driven image enhancement method (Luo, 2024). Liu et al. (2025a) introduced a framework that cascades an aerial image enhancement module with AC3Net, while Xiao et al. (2024) proposed a neuromorphic computing-based underwater image enhancement network (UIEN), which simulates visual system perception and employs unsupervised learning to address multiple types of underwater image degradation and validate its effectiveness. Despite these significant advances in image enhancement, most existing studies still overlook data privacy issues, as unencrypted images transmitted over networks are vulnerable to theft or misuse. This further highlights the necessity of integrating image encryption with recognition.

Therefore, with increasing emphasis on data privacy, researchers have begun integrating image encryption with disease detection to achieve end-to-end security without compromising performance. Qin et al. (2014) proposed SecSIFT, a method that performs SIFT feature extraction directly within the encrypted domain, effectively safeguarding sensitive image data while maintaining high detection accuracy and computational efficiency. Building on this idea, Man et al. (2021) integrated convolutional neural networks with chaotic encryption, enabling intelligent privacy protection for both image and text data, and laying the foundation for secure image processing in agriculture. Kumar et al. (2021) introduced SP2F, a privacy-preserving framework combining blockchain and deep learning, with a two-level privacy engine and stacked LSTM networks to improve UAV data authentication and resilience. Furthermore, Kethineni and Gera (2023) proposed an IoT security model that integrates sparse capsule autoencoders and attention-based GRUs for lightweight detection and data protection, achieving 99.9% accuracy and F1 score, highlighting its potential for agricultural data security.

Our work aims to develop a lightweight deep learning model for precise crop disease detection and robust image-level privacy protection. Optimized for resource-constrained edge devices, it ensures real-time, high-precision identification of various disease types. Additionally, to secure data transmission, we integrate a novel image encryption scheme based on a 3D chaotic cube, effectively preventing unauthorized access without compromising detection performance. Our model has been comprehensively evaluated on real-world datasets and outperforms existing methods in detection accuracy, computational overhead, and privacy protection. This solution offers a practical and secure pathway for smart agriculture applications. Our approach addresses two key challenges in plant disease detection: data privacy and detection accuracy.

**Figure 1:**
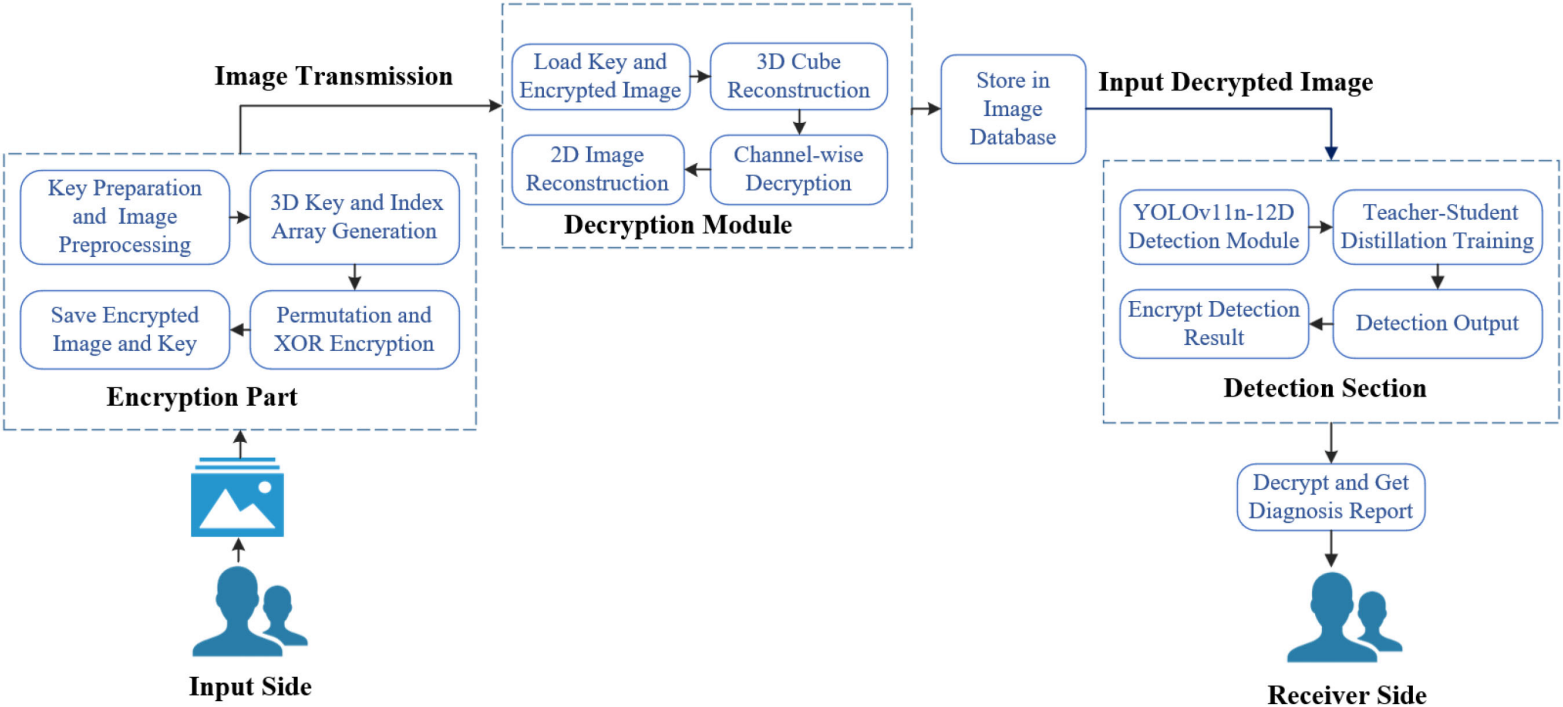
Overview of the process.

Our main contributions are as follows:

- We propose an encryption model combining SHA-256, a 3D Logistic Map, pixel permutation, and XOR operations, ensuring both strong security and high efficiency. Compared to traditional Advanced Encryption Standard(AES) and Rivest-Shamir-Adleman(RSA), our method offers a larger key space, enhanced attack resistance, and millisecond-level encryption speeds, making it well-suited for edge and mobile devices in agriculture. Security evaluations using entropy, Structural Similarity Index Measure(SSIM), Number of Pixel Change Rate(NPCR), and Unified Average Changing Intensity(UACI) confirm its balanced performance.
- We present a knowledge distillation framework with YOLOv12s as the teacher and YOLOv11n as the student. The distilled student model, YOLOv11n-12D, inherits enhanced detection capabilities while maintaining a lightweight structure. To address class imbalance and improve small lesion detection, Focal Loss and CIoU Loss are incorporated during training. Experimental results show that YOLOv11n-12D outperforms existing lightweight models in precision, recall, F1 score, and mAP, while achieving real-time inference speed.
- We develop an end-to-end system in which farmers encrypt images locally, transmit them wirelessly to a diagnostic center, and receive encrypted detection results. This framework ensures data security and scalability across various crop scenarios, effectively integrating deep learning and encryption technologies. The overall architecture is shown in **Figure 1**.

The rest of this paper is organized as follows: Section 2 reviews related work. Section 3 details the encryption method. Section 4 introduces the detection model. Section 5 describes data processing and optimization. Section 6 presents experiments and analysis. Section 7 concludes the paper.Section 8 highlights the system’s significance, practical value, and limitations.

## 2 Related work

Traditional encryption algorithms such as AES and DES are inadequate for real-time protection of agricultural images due to high dimensionality, redundancy, and computational overhead associated with such data. Although deep learning has achieved success in plant disease detection tasks, most existing studies overlook privacy concerns during image transmission and processing. To contextualize the proposed integrated system, this section reviews key image encryption techniques and plant disease detection approaches.

### 2.1 Data encryption techniques

In response to the need for secure image transmission in agriculture, several encryption techniques have been developed, each aiming to balance security and efficiency. Key representative methods are summarized below. Niyat et al. (2017) proposed an encryption scheme based on non-uniform cellular automata (CA) and hyper-chaotic mapping, enhancing key space and attack resistance. Kulalvaimozhi et al. (2020) introduced a method combining homomorphic encryption (NHE) and enhanced discrete wavelet transform (EDWT), improving both security and compression efficiency. Priyanka et al. (2024) employed 3D chaotic mapping and Huffman coding for medical image encryption. Sha et al. (2024) developed an IoT-oriented image encryption scheme utilizing graph data structures and logic gate mechanisms to strengthen attack protection. Ding et al. (2022) proposed a GAN-based key generation model, significantly improving key security.

Devi et al. (2024) proposed a DWT-SVD watermarking and PSMD symmetric encryption scheme to enhance UAV image security. While effective, its reliance on symmetric keys may pose challenges in key management and attack resistance. Zhou et al. (2024) applied compressed sensing and a two-dimensional hyperchaotic coupled Fourier oscillator system (2D-HCFOS) to improve encryption speed and security, achieving promising simulation results. Chen et al. Zhou et al. (2025) introduced a 2D super-attractor Logistic coupled chaotic model (2D-SALC), outperforming existing methods in chaos and security metrics. However, further validation, including integration with YOLO models and assessment of encryption impact on detection accuracy, remains needed.

### 2.2 Deep learning-based disease monitoring

Deep learning has shown strong results in plant disease detection Attri et al. (2023), with notable performance across crops like rice, wheat, tomato, and grape. Jia et al. (2023) improved YOLOv7 for rice pest detection by integrating MobileNetV3 and coordinate attention, achieving 92.3% accuracy and 93.7% mAP@0.5. However, its performance in complex backgrounds still faces challenges. To address this, Deng et al. (2023) enhanced YOLOv5s and YOLOv7-tiny models for better accuracy and speed, enabling mobile deployment. Liu and Wang (2020) optimized YOLOv3 with an image pyramid for better multi-scale detection in tomato disease recognition. Zhang et al. (2023) proposed RYWD and SSA networks for wheat Fusarium head blight detection, improving accuracy and precision by 11.8% and 10.7%. Wu et al. (2023) combined YOLOv5 with HRNet for grape stem localization, achieving 92% accuracy in bunch detection and 90.2% in stem recognition. While these methods show improvements, their performance under complex field conditions still requires further refinement.

For eggplant disease detection, Liu et al. (2025b) enhanced YOLOv8n with the YOLO-RDM model, improving accuracy and robustness. Huang et al. (2024) proposed YOLOv8-E, which enhanced detection accuracy and small target recognition while reducing computational complexity. MR et al. Haque and Sohel (2022) used a dual-stream architecture combining CNN-SVM and CNN-Softmax, outperforming traditional models. Despite these advances, challenges remain in achieving high accuracy, robustness, and data security.

Despite significant progress in image encryption and plant disease detection, several critical gaps remain. Most existing studies treat encryption and detection as separate processes, lacking a unified solution that simultaneously ensures privacy protection and detection accuracy. Moreover, few works consider the resource constraints of real-time processing on edge devices. Many YOLO-based methods either overlook the impact of encryption on feature extraction or employ models that are too heavy for mobile deployment. Therefore, there is a need for a unified lightweight framework that guarantees image security while enabling efficient disease detection. To address this gap, we propose an integrated system that combines 3D chaotic cube encryption with the YOLOv11n-12D detection model, aiming to enhance both detection performance and data security.

## 3 3D chaotic cube encryption scheme

**Figure 2:**
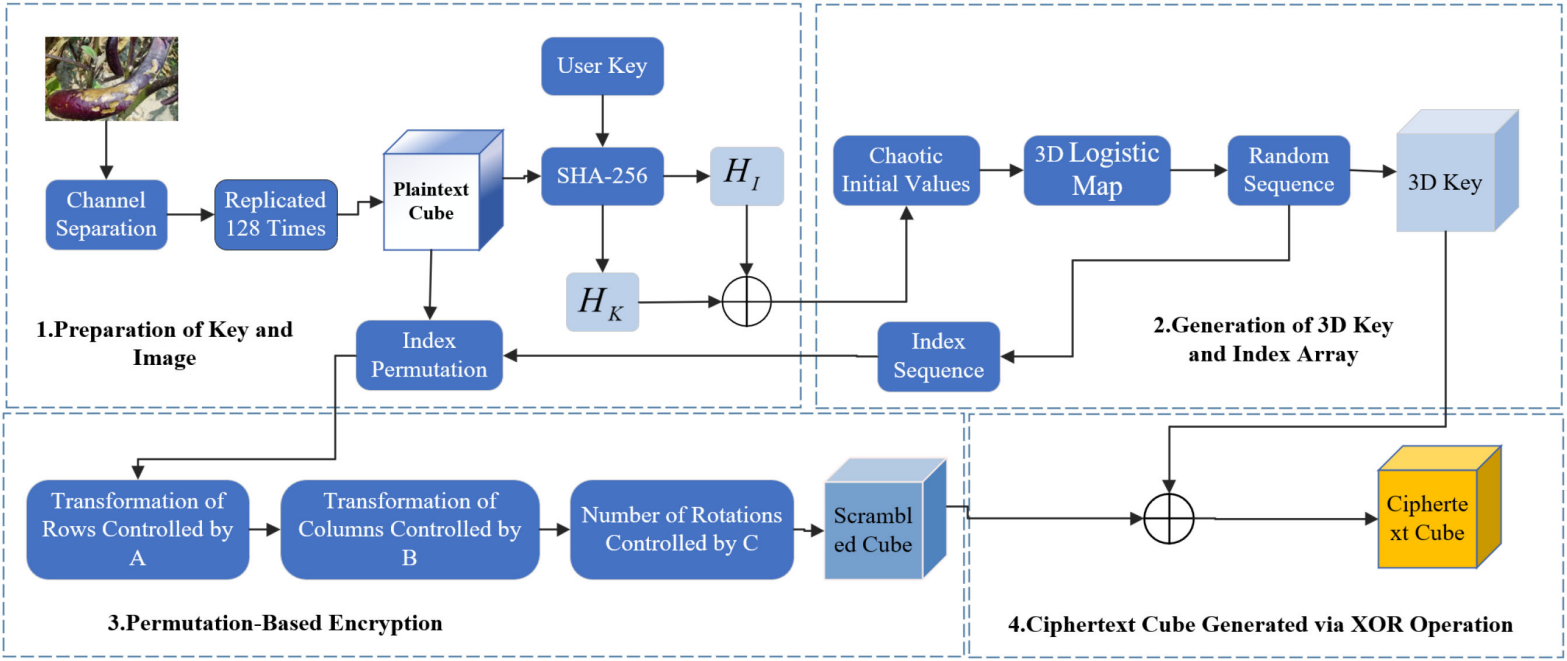
Encryption flowchart.

**Figure 3:**
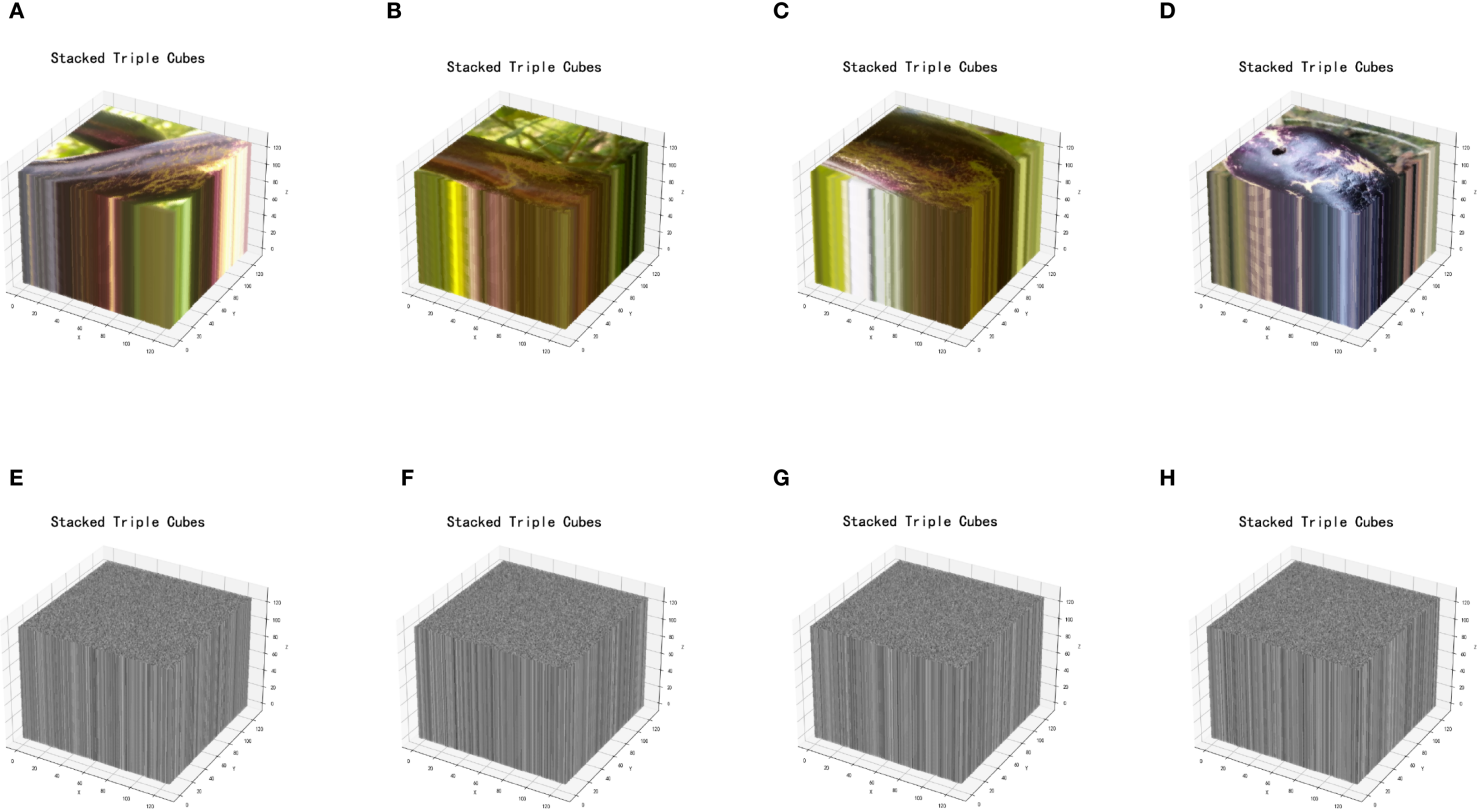
**(A-D)** show the original images, **(E-H)** display the encrypted images.

In eggplant disease detection, image encryption is essential for data security by preventing unauthorized access, tampering, and maintaining integrity. Ciphertext transmission enhances system security and reduces the risk of cyberattacks. This section presents a novel image encryption method based on a 3D Chaotic Cube Encryption Scheme, which consists of four steps: preparation of the key and image, generation of 3D key and index array, permutation encryption and XOR operation, and save the encrypted image and key. Compared to frequency- and chaos-based methods (Jui-Cheng and Guo, 2000; Armand Eyebe Fouda et al., 2014; Jammula et al., 2022), the proposed scheme offers stronger resistance to attacks and superior performance. **Figure 2** illustrates the encryption framework, and **Figure 3** shows the original and encrypted eggplant images.

### 3.1 Preparation of the key and image

Prior to encryption, a 64-bit hexadecimal key and the target image are provided. The key is then processed using a hash function to generate the initial values for the chaotic system. The provided 64-bit hexadecimal string key hex is first converted into binary, and its SHA-256 hash value is computed: H_K_=SHA-256(key_hex). Assume the original image *img* has a size of 128 × 128. Extract the R, G, and B channels separately as R(*i,j*), G(*i,j*), and B(*i,j*), where *i,j* ∈ {0,127}. The result is as shown in Equations 1–3. Then expand the image into a 128 × 128 × 128 3D cube and *K* ∈ {0,127}:


(1) $$C_{R}(i,j,k)=R(i,j)$$

(2) $$C_{G}(i,j,k)=G(i,j)$$

(3) $$C_{B}(i,j,k)=B(i,j)$$

The three channel cubes are concatenated into a one-dimensional bitstream. The image hash is then computed as shown in Equation 4:


(4) $$H_{I}=\text{SHA−}256(\text{img})$$

To initialize the chaotic system, extract the first, middle, and last 64 bits from the bitstream, convert them into decimal values *x*_0_ *,y*_0_ *,z*_0_, and normalize each to the range (0,1). These values are used as initial conditions for the chaotic system, x_0_,y_0_,z_0_ as defined in Equations 5–7:


(5) $$x_{0}=\frac{\text{int}(H_{K}[0:16],16)}{2^{64}}$$

(6) $$y_{0}=\frac{\text{int}(H_{K}[16:32],16)}{2^{64}}$$

(7) $$z_{0}=\frac{\text{int}(H_{K}[48:64],16)}{2^{64}}$$

### 3.2 Generation of 3D key and index array

- To generate chaotic sequences, the 3D Logistic Map is employed with the following initial conditions, as defined in Equations 8–10:

(8) $$x_{1}={\text{r}}_{x}\cdot x_{0}\cdot (1-x_{0})+\beta \cdot y_{0}\cdot z_{0}$$

(9) $$y_{1}={\text{r}}_{y}\cdot y_{0}\cdot (1-y_{0})+\gamma \cdot x_{0}\cdot z_{0}$$

(10) $$z_{1}={\text{r}}_{z}\cdot z_{0}\cdot (1-z_{0})+\alpha \cdot x_{0}\cdot y_{0}$$

Then use *x*_1_ *, y*_1_ *, z*_1_ in the equations again to calculate the next values of *x*_2_ *,y*_2_ *,z*_2_ repeat this process to generate a chaotic sequence array as shown in Equations 11–13:


(11) $$x_{n+1}={\text{r}}_{x}\cdot x_{n}\cdot (1-x_{n})+\beta \cdot y_{n}\cdot z_{n}$$

(12) $$y_{n+1}={\text{r}}_{y}\cdot y_{n}\cdot (1-y_{n})+\gamma \cdot x_{n}\cdot z_{n}$$

(13) $$z_{n+1}={\text{r}}_{z}\cdot z_{n}\cdot (1-z_{n})+\alpha \cdot x_{n}\cdot y_{n}$$

**Figure 4:**
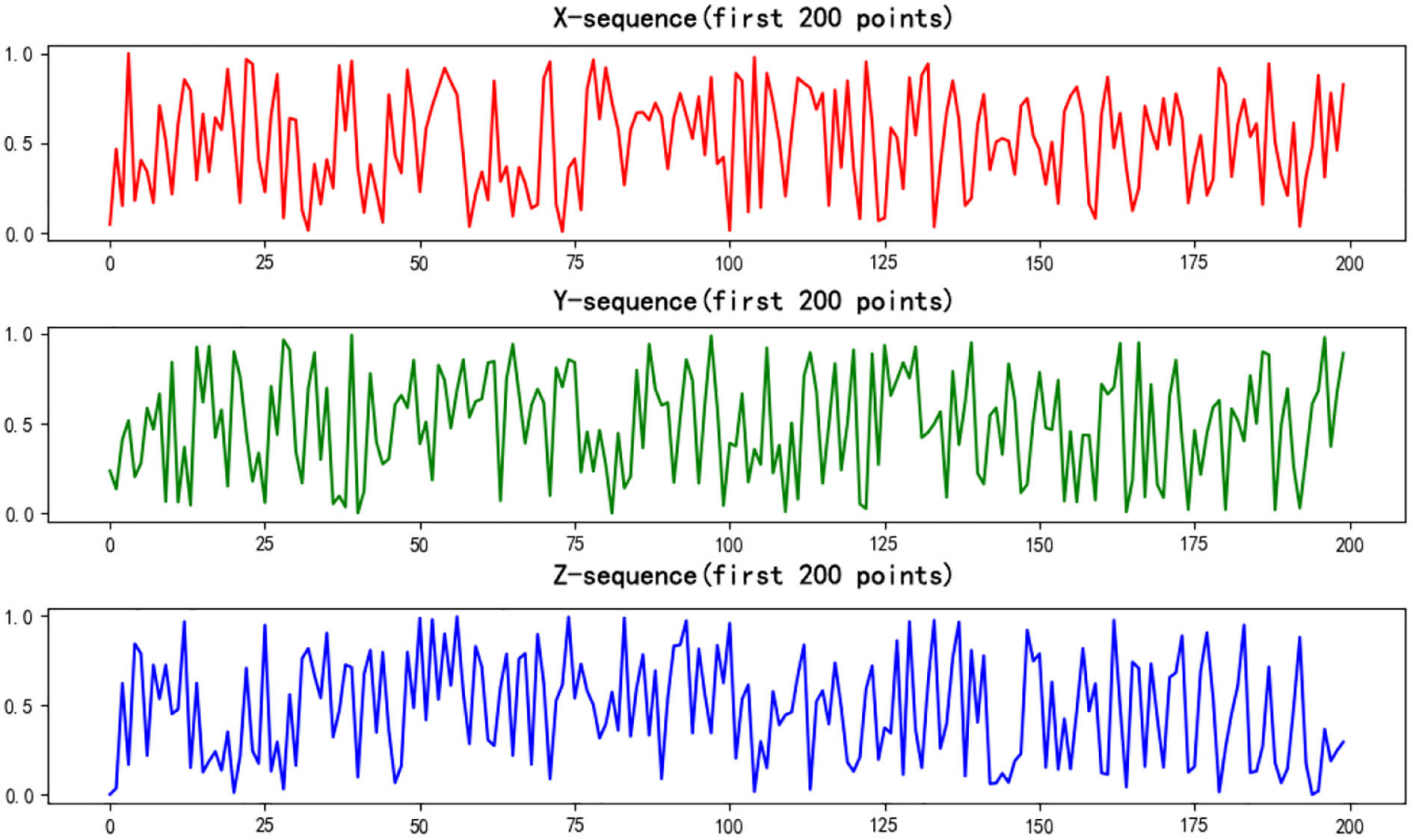
Random sequence numbers.


Here, *r_x_,r_y_,r_z_* ∈ (3, 57, 4), Take the values from the chaotic interval. These *α, β, γ* control the coupling degree of the system. The 3D Logistic Map is iterated one million times, and the initial steps are discarded to eliminate transient effects. This process generates three long chaotic sequences. **Figure 4** shows the resulting random sequences, chaotic sequences as defined in Equations 14–16:

(14) $$X=\{x_{1},x_{2},\ldots ,x_{N}\}$$

(15) $$Y=\{y_{1},y_{2},\ldots ,y_{N}\}$$

(16) $$Z=\{z_{1},z_{2},\ldots ,z_{N}\}$$

Map the values to the range [0, 255] to form the 3D key: *K*(*x,y,z*)= [*X*(*x,y,z*) × 256].

- To construct the index arrays, X is sorted to obtain A = argsort(X), and Y is sorted to obtain B = argsort(Y), Z is normalized to the range [0, 3], which is used for rotation: C = [Z × 4].

### 3.3 Permutation encryption and XOR operation

- First, apply index-based permutation to the 3D cube using arrays A and B to reorder rows and columns, respectively. Specifically, perform row permutation as: $C_{R}\to (:,A,:)$ , $C_{G}\to (:,A,:)$ , $C_{B}\to (:,A,:)$ , and column permutation: $C_{R}\to (B,:,:)$ , $C_{G}\to (B,:,:)$ , $C_{B}\to (B,:,:)$ . and rotation based on the value of C , rotate each layer C(i,j) times $90^{\circ }$ . The result as shown in Equations 17–19:

(17) $$C_{R}(:,:,i)=\text{Rotate}(C_{R}(:,:,i),C(i,j))$$

(18) $$C_{G}(:,:,i)=\text{Rotate}(C_{G}(:,:,i),C(i,j))$$

(19) $$C_{B}(:,:,i)=\text{Rotate}(C_{B}(:,:,i),C(i,j))$$

- Next, complete the encryption by performing a bitwise XOR operation between the permuted 3D cube and the 3D key: ${C^{\prime }}_{R}=C_{R}⊕K,\;{C^{\prime }}_{G}=C_{G}⊕K,\;{C^{\prime }}_{B}=C_{B}⊕K$

### 3.4 Save the encrypted image and key

The encrypted 3D cube is converted back into a 2D color image by mapping the corresponding values to the RGB channels as shown in Equations 20–22:


(20) $$R^{\prime }(i,j)=C^{\prime }{\;}_{R}(i,j,0),$$

(21) $$G^{\prime }(i,j)=C^{\prime }{\;}_{G}(i,j,0),$$

(22) $$B^{\prime }(i,j)=C^{\prime }{\;}_{B}(i,j,0)$$

These channels are combined to generate the final encrypted image enc_img, which is saved to a file. The index arrays A, B, C, along with the key file key_bin, are stored for decryption.

### 3.5 Decryption process

To enable proper visualization and subsequent detection, the encrypted image must be decrypted. The decryption process involves four steps: load the key and encrypted image, reconstruct the 3D cube, decrypt each channel, and rebuild the 2D image.

#### 3.5.1 Load the key and encrypted image

The decryption process begins by loading the 3D key and index arrays (A, B, C) from the file system, along with the encrypted image file.

#### 3.5.2 Reconstruct the 3D cube

Each color channel (R, G, B) of the image is reshaped into a 128 × 128 × 128 3D cube, where the first two dimensions represent pixel positions and the third dimension corresponds to the stacked image layers.

#### 3.5.3 Decrypt each channel

For each color channel’s 3D cube, two operations are applied:

- Bitwise XOR Restoration: The cube is first restored by applying a bitwise XOR operation with the original 3D key used during encryption.
- Reverse Rotation: Then, reverse rotations are performed based on the index arrays A and B. Array A controls the reversal along rows, and B controls the columns. The rotation direction is opposite to the encryption process. The number of 90° rotations is determined by the values in array C, applied in reverse order to maintain symmetry.

#### 3.5.4 Rebuild the 2D image

The decrypted 3D cubes of the R, G, and B channels are converted back into 2D images. These channels are then merged to reconstruct the final color image, which is output as the decrypted result.

## 4 YOLOV11N-12D model

**Figure 5:**
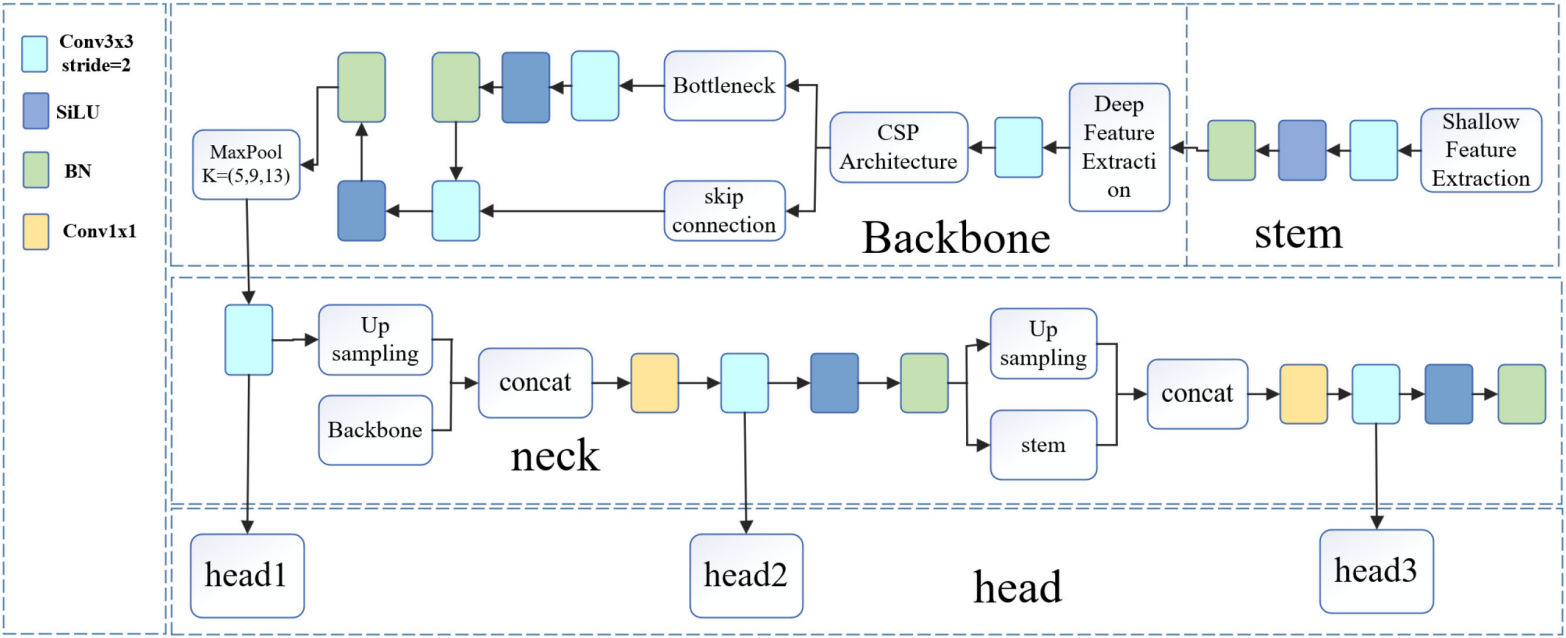
Detection model architecture.

**Figure 6:**
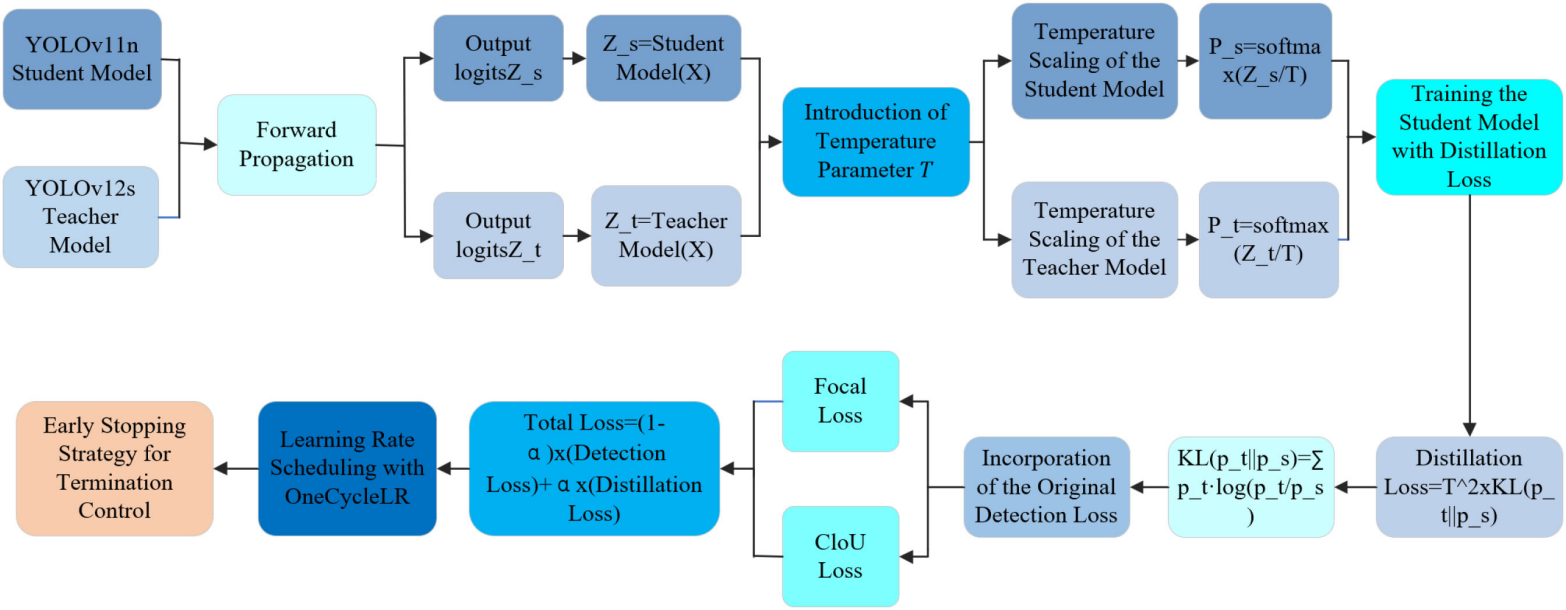
Knowledge distillation process flow.

Deep learning-based object detection has demonstrated remarkable performance in disease recognition, with the YOLO series widely used for its speed and accuracy (Dai et al., 2022; Wang et al., 2024; Xie et al., 2024). However, traditional YOLO models struggle when dealing with small objects and class imbalance (Obu et al., 2023; A.N. and A.P., 2022). To address these issues, we propose an enhanced lightweight model, YOLOv11n-12D. As shown in **Figure 5**, the architecture consists of four components: Stem, Backbone, Neck, and Head. In our model, YOLOv11n serves as the student model, while YOLOv12s acts as the teacher. By leveraging knowledge distillation and detection loss, we enhance recall, reduce missed detections, and maintain efficiency, making it suitable for large-scale agricultural applications. The distillation process is detailed in **Figure 6**.

The following are the steps of the distillation process:

a. The pre-trained YOLOv11n is used as the student model, and YOLOv12s as the teacher. Z_*t* and *Z*_s are defined as shown in Equations 23, 24: Augmented samples are input into both models to compute their respective logits:

(23) $$Z_{t}=\text{TeacherModel}(X)$$

(24) $$Z_{s}=\text{StudentModel}(X)$$

Since logits vary significantly in magnitude, a temperature parameter *T = *4.0 is applied to smooth them, stabilize gradients, and generate soft labels for distillation.

b. Temperature scaling (*T = *4.0) is applied to smooth the logits and obtain softened probability distributions for effective knowledge transfer as shown in Equations 25, 26:

(25) $$P_{t}=\text{softmax }(\frac{Z_{t}}{T})$$

(26) $$P_{s}=\text{softmax }(\frac{Z_{s}}{T})$$

When *T = *1.0, the distribution reduces to standard softmax, limiting the ability to learn from the teacher. We adopt Kullback-Leibler divergence as the distillation loss: Distillation Loss = *T* ^2^ × KL(p *_t_* ∥ p*_s_*). Here, *T* ^2^ offsets the gradient scaling effect caused by smoothing. A smaller KL value indicates better alignment between the student and teacher outputs, KL as defined in Equation 27:


(27) $$\text{KL}(pt∥ps)=\sum i{\left(pt\right)}_{i}\cdot \text{log }(\frac{(pt)i}{(ps)i})$$

c. The student model is trained to balance knowledge from the teacher and performance on the original task. To achieve this, the detection loss and distillation loss are combined with a weighted sum: TotalLoss = (1 − *α*) × (Detection Loss) + *α* × (Distillation Loss). Mixed precision training, learning rate scheduling, and early stopping strategies are employed to improve efficiency and convergence.
d. The detection loss consists of a weighted sum of classification loss (Focal Loss) and regression loss (CIoU Loss):Detection Loss=Focal Loss + CIoU Loss, A weight of *α = *0.7 is used to emphasize the distillation loss. The learning rate is adjusted using the OneCycleLR policy as shown in Equation 28:

(28) $$\text{LR}(t)=\{\begin{array}{ll} {\text{LR}}_{\text{max}}\times \frac{t}{t_{up}} & if\;t\leq t_{\text{up}}\\ {\text{LR}}_{\text{max}}\times (1-\frac{t\;-\;t_{up}}{t_{down}}) & if\;t>t_{\text{up}} \end{array}$$

e. Training is terminated using an early stopping strategy when the condition specified in Equation 29 is met.

(29) if Patience Count≥10,stop training

After each epoch,the validation set is evaluated using mAP, Precision, and Recall to monitor the effectiveness of the distillation strategy on student model performance.

## 5 Materials preparation and optimization methods

**Figure 7:**
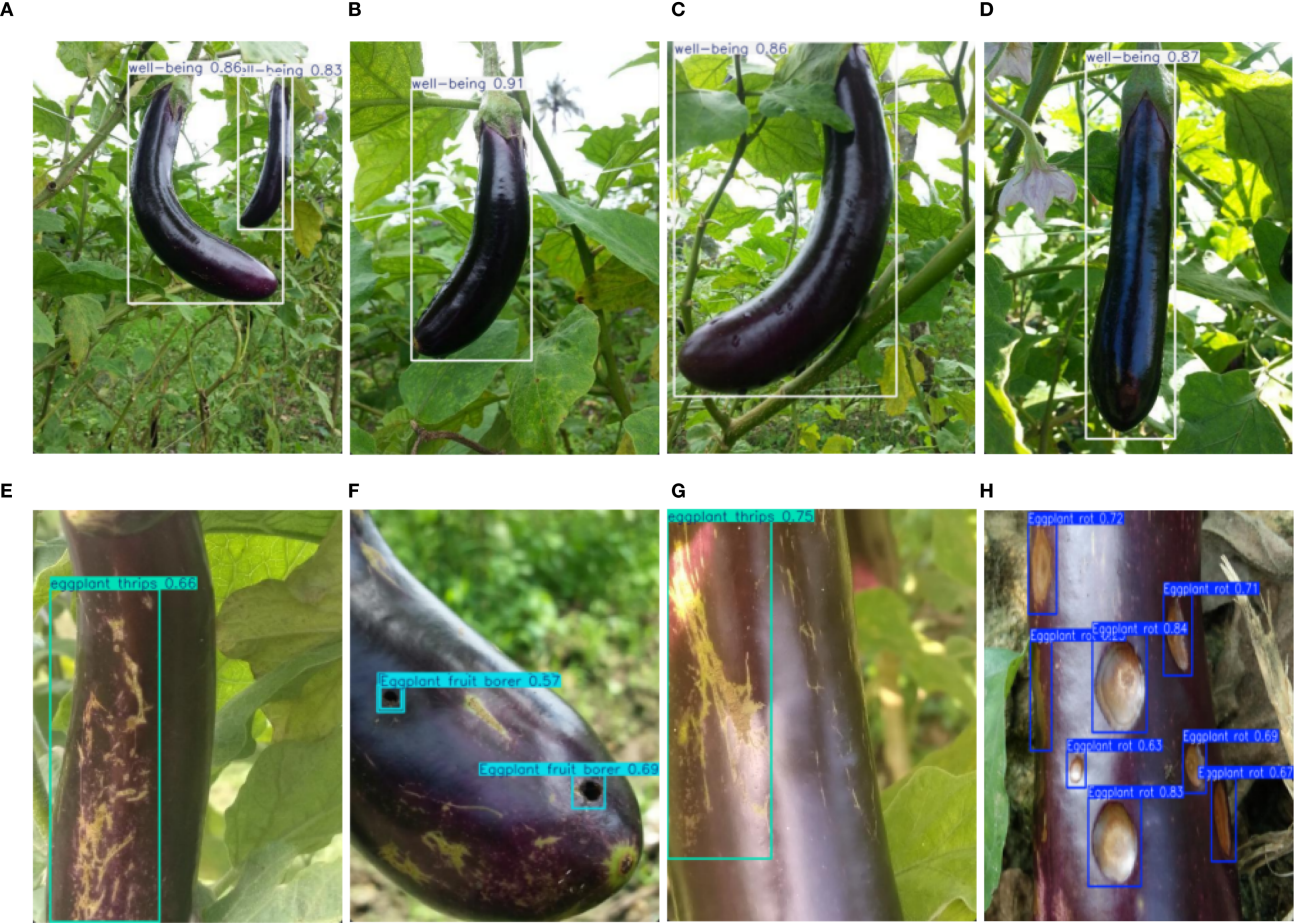
**(A-D)** are healthy eggplant images, **(E-H)** are diseased eggplant images.

**Figure 8:**
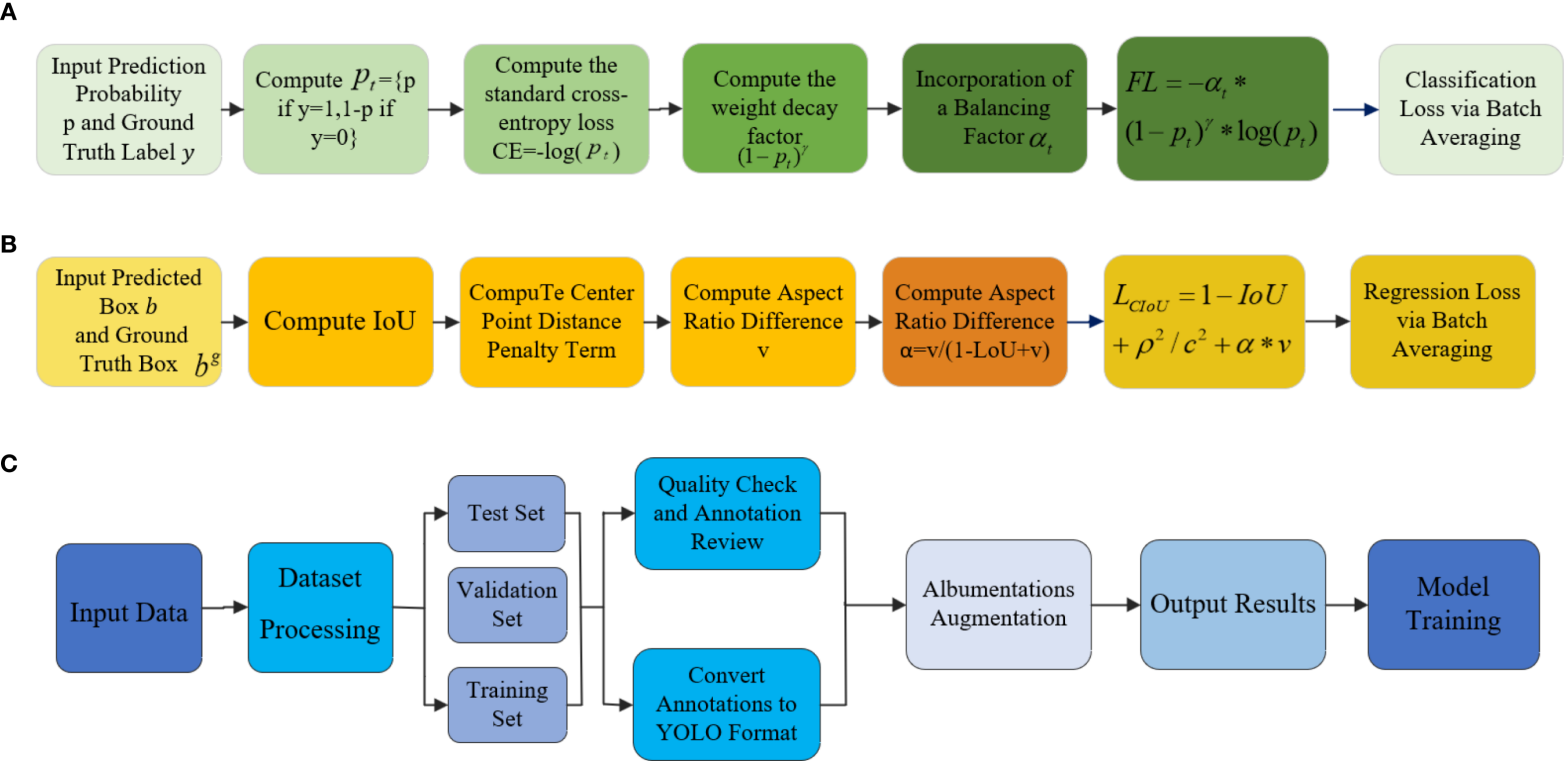
Diagram of materials analysis and optimization. **(A)** Focal loss. **(B)** CIoU loss. **(C)** Material preparation and optimization.

To ensure high efficiency and accuracy in eggplant disease detection, a large-scale dataset was constructed, encompassing four categories: eggplant rot, fruit borer, healthy samples, and thrips. All images were annotated in YOLO format with standardized bounding boxes and precise class labels. The annotations were reviewed by agricultural experts to ensure high-quality and consistent labeling.The dataset was collected from multiple eggplant cultivation bases, encompassing various growth cycles, diverse lighting conditions, and all developmental stages of pest/disease infestation (from initial infection to characteristic symptom manifestation). Specifically, the test set comprises 745 representative images (containing 1516 annotated instances), while the remaining 7520 images were partitioned into training (5264 images) and validation (2256 images) sets at a 7:2:1 ratio. This scientifically designed partitioning scheme ensures both sufficient training data volume and reliable evaluation of model generalization capability. Sample differences between healthy and diseased eggplants are shown, highlighting the visual variability between categories (**Figure 7**). To address the limited quantity and variable quality of the collected raw images, we employed the Albumentations data augmentation library to enhance dataset diversity and improve model generalization and robustness (Buslaev et al., 2020; Korra et al., 2022). The overall workflow for material preparation and optimization is illustrated (**Figure 8**), and the specific data augmentation strategies are detailed as follows:

- Random Flip: Applies horizontal and vertical flips to simulate different viewing angles, enhancing feature recognition and robustness.
- Color Jitter: Alters brightness, contrast, and saturation to mimic various lighting conditions.
- Random Crop: Generates new samples by cropping image regions, helping reduce reliance on specific areas.
- Random Rotation: Rotates images within ±30° to improve viewpoint diversity and reduce angle bias.
- Random Noise Addition: Introduces Gaussian or salt-and-pepper noise to improve performance under degraded image conditions.
- Mosaic Augmentation: Merges multiple images to enrich contextual and visual diversity in complex scenes.
- MixUp Augmentation: Blends two images and labels to promote smooth label transition and mitigate overfitting.

The applied augmentation techniques significantly enhance the model’s robustness and generalization, allowing more reliable recognition of eggplant disease features under diverse conditions. Furthermore, the integration of Focal Loss and CloU Loss improves detection accuracy, achieving a final accuracy of 99.1% and effectively reducing the miss detection rate, thereby improving applicability in real-world agricultural scenarios.

### 5.1 Focal loss

Focal Loss is designed to address class imbalance, especially in single-stage detectors like RetinaNet. Traditional cross-entropy is dominated by easy negatives, causing unstable training. Focal Loss introduces a modulation factor to focus learning on hard examples, improving detection of minority classes.The construction of this function is shown in part (a) of **Figure 8**. For a binary label: *y* ∈ {0,1}(0 for positive class and 1 for negative class), the predicted probability pt is defined as Equation 30:


(30) $$p_{t}=\{\begin{array}{ll} p & if\;y=1\\ 1-p & if\;y=0 \end{array}$$

The standard cross-entropy loss is: CE = −log(p*_t_*), Calculate the weight decay factor (1 − p*_t_*)*^γ^*, *γ* is Hyperparameters, Typically takes values of 2 or 3, when *p_t_* is small,the decay factor approaches 1 when p is small; otherwise, it approaches 0. A balancing factor is introduced to control the ratio of positive to negative samples. The final Focal Loss as presented in Equation 31:


(31) $$\text{FL}=-\alpha (1-p_{t}{)}^{\gamma }\text{ log }(p_{t})$$

To compute the average Focal Loss for all samples in a batch, the classification loss is: $Focal\;Loss=\frac{1}{N}\sum_{i=1}^{N}FL(p_{t}^{\left(i\right)})$

### 5.2 CIoU loss

CIoU Loss is primarily used for object bounding box regression, especially in the context of rotated object detection. It optimizes the accuracy of the bounding box’s location, size, and angle. CIoU Loss was introduced to improve the detection accuracy of rotated objects, as traditional bounding box regression methods typically only consider rectangular boxes, while CloU Loss also accounts for the angle of the rotated boxes. CloU Loss optimizes the bounding box regression of object detection models by considering the center point error, size error (width and height), and rotation angle error. The construction of this function is shown in part (b) of **Figure 8**. Calculate the Intersection over Union (IoU) between the predicted box b and the ground truth box *b^g^*. As shown in Equation 32:


(32) $$\text{IoU}=\frac{Intersection\;Area}{Union\;Area}$$

Calculate the Euclidean distance between the center of the predicted box and the center of the ground truth box, as defined in Equation 33:


(33) $$\rho^{2}(b,b^{g})={\left(x-x^{g}\right)}^{2}+{\left(y-y^{g}\right)}^{2}$$

c can represent the diagonal distance of the smallest enclosing box that contains both the predicted box and the ground truth box as shown in Equation 34: Distance penalty term:


(34) $$\frac{\rho^{2}(b,b^{g})}{c^{2}}$$

Measure the difference in aspect ratio between the predicted box and the ground truth box, as defined in Equation 35:


(35) $$V=\frac{4}{\pi^{2}}{\left(\text{arctan }\frac{w^{g}}{h^{g}}-\text{arctan }\frac{w}{h}\right)}^{2}$$

Adaptive weight *α* is primarily used to balance the contribution of the aspect ratio loss term v in the overall CIoU loss. The result as shown in Equation 36:


(36) $$\alpha =\frac{v}{(1-IoU)+v}$$

By combining IoU, center distance, and aspect ratio, the final CIoU Loss is obtained as shown in Equation 37:


(37) $$L_{\text{CIoU}}=1-\text{IoU}+\frac{\rho^{2}(b,b^{ɡ})}{c^{2}}+\alpha v$$

## 6 Performance analysis

This section presents the evaluation metrics used to assess the performance of the two core components of the proposed system: the 3D chaotic cube-based encryption scheme for image security, and the YOLOv11n-12D-based detection model for eggplant disease diagnosis.

### 6.1 Analysis of the proposed encryption scheme

To evaluate our encryption scheme, we developed a quantitative assessment system for image encryption security (**Table 1**). It uses seven key indicators: contrast and mean square error (positively correlated) indicate pixel perturbation; information entropy shows randomness; while structural similarity (SSIM), energy value, homogeneity, and structural content (negatively correlated) assess structural damage, pattern concealment, and pixel disorder. This framework is based on research by Gupta and Chauhan (2021); Rahman et al. (2025), and Karmakar et al. (2021).

**Table 1:** Evaluation parameters and their relation with image encryption security.

P.	M.E.	R.W.S.S.	V.E.
Contrast	$\text{Contrast}=\sum_{a,b}|a-b{|}^{2}\;O(a,b)$	Contrast ∝ S.S	Higher contrast reduces predictability and enhances security.
SSIM	$SSIM=(2\mu_{x}\mu_{y}+C_{1})(2\sigma_{xy}+C_{2})/(\mu_{x}^{2}+\mu_{y}^{2}+C_{1})(\sigma_{x}^{2}+\sigma_{y}^{2}+C_{2})$	$\text{SSIM}\propto \frac{1}{\text{S}.\text{S}}$	Lower SSIM prevents structural leakage, improving security.
MSE	$MSE=\frac{1}{N}\sum_{i=1}^{N}{\left(I_{1}(i)-I_{2}(i)\right)}^{2}$	MSE ∝ S.S	Higher MSE increases difference, making decryption harder.
Entropy	$\text{Entropy}=-\sum_{i=0}^{\mathrm{255}}p_{i}\times \log_{2}(p_{i})$	Entropy ∝ S.S	Higher entropy means more randomness, improving security.
Energy	$\text{Energy}=\underset{a,b}{\sum }{\left[O(a,b)\right]}^{2}$	$\text{Energy}\propto \frac{1}{\text{S}.\text{S}}$	Lower energy hides patterns, strengthening security.
Homogeneity	$\text{Homogeneity}=\underset{a,b}{\sum }\frac{O(a,b)}{1+|a-b|}$	$\text{Homogeneity}\propto \frac{1}{\text{S}.\text{S}}$	Lower homogeneity increases pixel chaos, improving security.
SC	$\text{SC}=\frac{\sum (\text{original }{\text{image}}^{2})}{\sum {\left(\text{original}-\text{encrypted}\right)}^{2}}$	$\text{SC}\propto \frac{1}{\text{S}.\text{S}}$	Lower SC means less similarity to the original, enhancing security.

P, Parameter; M.E., Mathematical Equation; R.W.S.S., Relationship with Strong Security (S.S); V.E., Variable Explanation.

**Table 2** shows that our method outperforms existing technologies (Huang et al., 2025; Xu et al., 2024; Ullah et al., 2025) in encryption quality, security, and efficiency. By integrating chaotic sequence generation, pixel permutation, and XOR encryption, our solution maintains consistent performance metrics for all test samples (both healthy and diseased eggplants). The entropy value is 7.6195 (close to the theoretical maximum of 8), and the pixel correlation coefficient is −0.0084 (close to 0). Our method achieves high image fidelity (40.26 dB) and fast encryption speed (0.0127 seconds), which is 23 times faster than the fastest comparative method. It also preserves key features for disease identification, meeting smart agriculture’s requirements for real-time performance, security, and feature preservation.

**Table 2:** Comparative performance analysis of eggplant image encryption methods.

Proposed work (encrypted images)								
Image type	Homogeneity	SC	Entropy	Correlation		Energy	Contrast	Execution time (s)
Healthy 1	0.0158	0.6381	7.6195	−0.0084		0.0001	4905.8639	0.0160
Healthy 2	0.0158	0.8239	7.6195	−0.0084		0.0001	4905.8639	0.0124
Healthy 3	0.0158	0.4249	7.6195	−0.0084		0.0001	4905.8639	0.0131
Healthy 4	0.0158	0.3750	7.6195	−0.0084		0.0001	4905.8639	0.0127
Diseased 1	0.0158	**0.9212**	7.6195	−0.0084		0.0001	4905.8639	**0.0111**
Diseased 2	0.0158	0.5680	7.6195	−0.0084		0.0001	4905.8639	0.0119
Diseased 3	0.0158	0.6245	7.6195	−0.0084		0.0001	4905.8639	0.0112
Diseased 4	0.0158	0.6458	7.6195	−0.0084		0.0001	4905.8639	0.0131
Mean	**0.0158**	**0.6277**	**7.6195**	**-0.0084**		**0.0001**	**4905.8639**	**0.0127**

Existing methods comparison								
Method	MSE	PSNR	Entropy	Correl.		Energy	Contrast	Execution Time (s)
15	5170.0723	10.9958	7.1346	−0.0707		0.0001	2706.7328	2.0395
41	8778.5593	8.6966	7.6232	0.2083		0.0001	3970.8332	0.2992
45	8256.4317	8.9629	**7.7475**	−0.0854		0.0000	**6158.4497**	0.8220
Ours	**0.158**	**40.2623**	7.6195	**-0.0084**		**0.0001**	4905.8639	**0.0127**

Bold values are representative or key results.

### 6.2 Key space analysis

The key space, representing the total number of possible keys, is a critical factor in resisting brute-force attacks. In this scheme, the user key is a 64-character hexadecimal string, corresponding to 256 bits. As each hex character encodes 4 bits, the key space size is: 2^256^ ≈ 1.16 × 10^77^. Such a vast key space is computationally infeasible to exhaust. Even at 10^18^ keys per second, a brute-force search would take: $\frac{1.16\times 10^{77}}{10^{18}\times 60\times 60\times 24\times 356}\approx 3.67\times 10^{50}$ year. These results confirm that the proposed key space is computationally infeasible to exhaust via brute-force attacks.

### 6.3 Attack resistance analysis

#### 6.3.1 Known-Plaintext attack

The proposed scheme uses a 3D Logistic Map, which exhibits strong sensitivity to initial conditions—tiny variations lead to drastically different outputs. The chaotic system evolves as shown in Equation 38:


(38) $$x_{n+1}=\text{r}\cdot x_{n}\cdot (1-x_{n})+\beta \cdot y_{n}\cdot z_{n}$$

Chaotic behavior is verified by the Lyapunov exponent, as defined in Equation 39:


(39) $$\lambda =\underset{n\to \infty }{\lim}\frac{1}{n}\sum_{i=1}^{n}\text{ln }|\frac{df(x_{i})}{dx_{i}}|$$

A positive exponent *λ >* 0, indicates exponential divergence. In our experiments, *λ = *0.89 confirms high sensitivity, making it extremely difficult to reverse-engineer the key, even with known plaintext–ciphertext pairs. This divergence is described as defined in Equation 40:


(40) $$\text{Δ}x_{n}=\text{Δ}x_{0}\cdot {\text{e}}^{\lambda n}$$

To initialize the chaotic system, we apply the SHA-256 hash function to the user key. With its strong collision resistance and irreversibility, the probability of a successful brute-force match is negligible, as defined in Equation 41:


(41) $$P=\frac{1}{2^{256}}$$

#### 6.3.2 Differential attack: NPCR

NPCR evaluates how a minor change in the input affects the encrypted output. It is defined as: $\text{NPCR}=\frac{\sum_{i,j}D(i,j)}{W\times H}\times 100\%$ . *D(i,j)* as defined in Equation 42:


(42) $$D(i,j)=\{\begin{array}{ll} 1 & if\;C_{1}(i,j)\neq C_{2}(i,j)\\ 0 & if\;C_{1}(i,j)=C_{2}(i,j) \end{array}$$

Here, C_1_(*i,j*) and C_2_(*i,j*) denote the pixel values of two encrypted images with slight input differences. The ideal NPCR approaches 100%.

#### 6.3.3 Differential attack: UACI

UACI quantifies the average intensity difference between two encrypted images, as shown in Equation 43:


(43) $$\text{UACI}=\frac{1}{W\times H}\underset{i,j}{\sum }\frac{\left|C_{1}(i,j)-C_{2}(i,j)\right|}{255}\times 100\%$$

**Figure 9:**
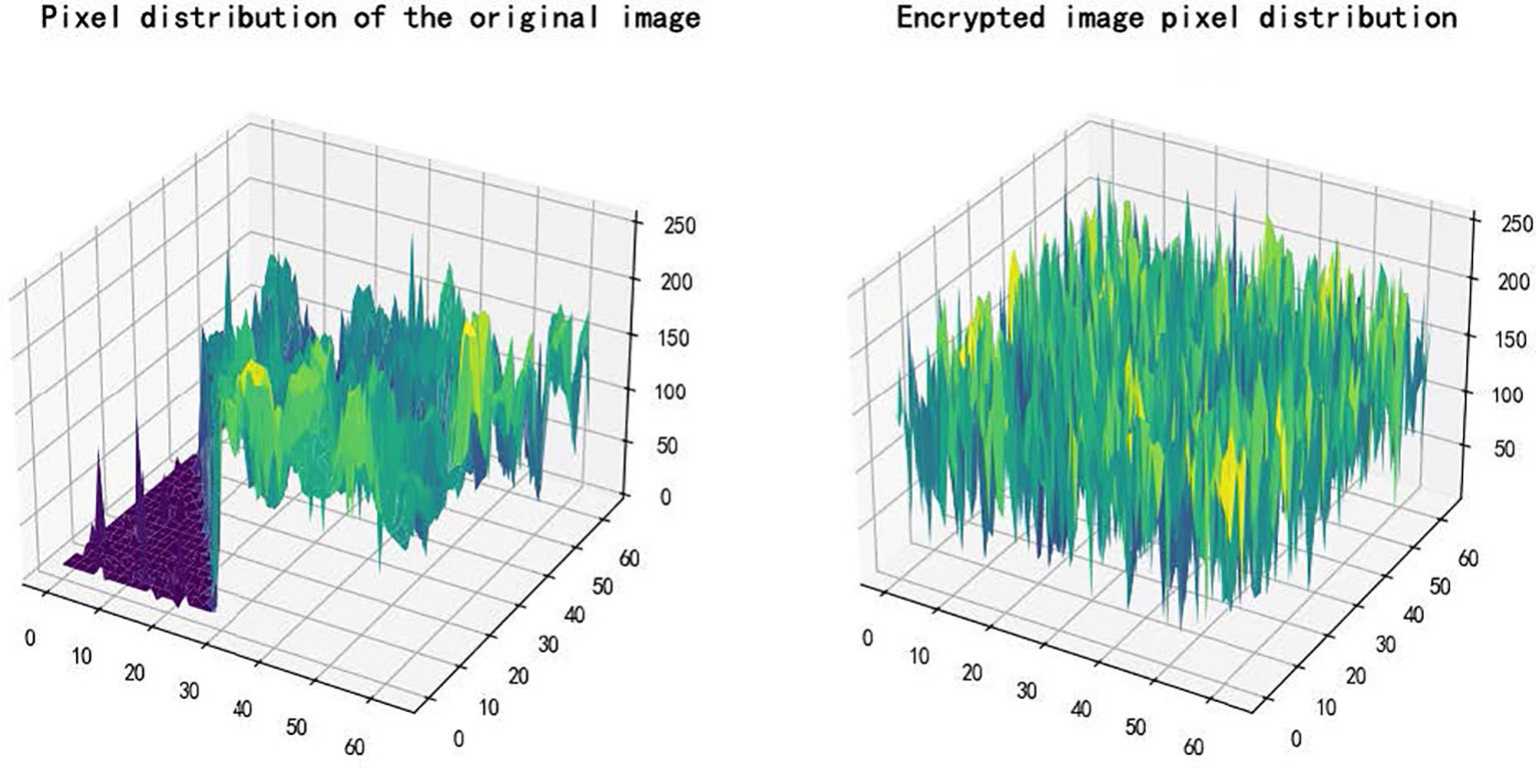
Pixel analysis diagram.

The ideal value should be close to 33%. Experimental results show: *NPCR = *99.63%*, UACI = *32.85%, These values confirm high resistance to differential attacks and strong sensitivity to input perturbations. **Figure 9** compares the pixel distribution: the original image (left) shows structured patterns, while the encrypted image (right) displays uniform randomness, demonstrating visual and statistical security.

### 6.4 Statistical analysis

#### 6.4.1 Histogram analysis

**Figure 10:**
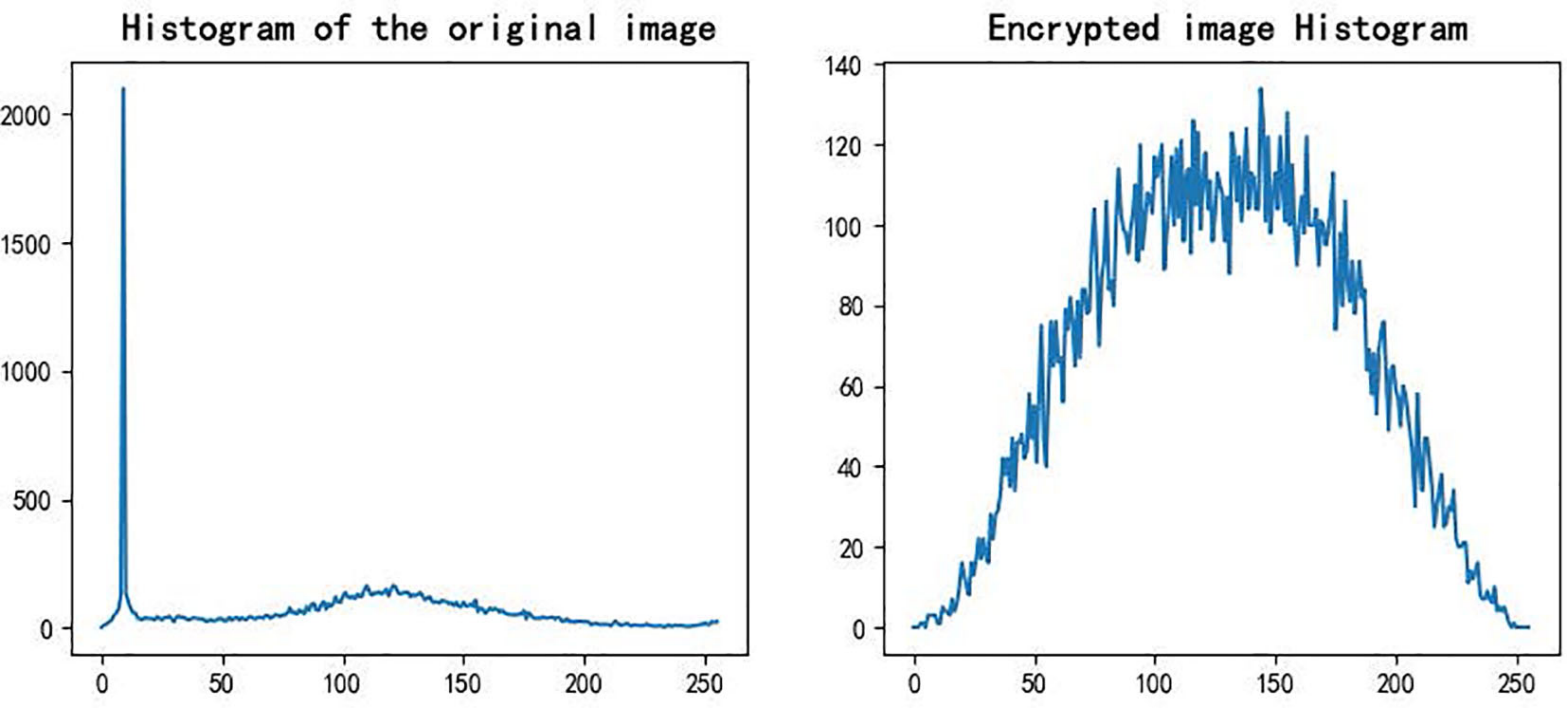
Image grayscale distribution.

**Figure 10** illustrates the grayscale distributions of the original and encrypted images. The original image shows a clear peak in pixel intensity, while the encrypted image exhibits a nearly uniform distribution with no apparent structure. This indicates that the encryption process effectively randomizes the statistical properties of the original image, eliminating pixel concentration and preventing histogram-based attacks.

#### 6.4.2 Pixel autocorrelation analysis

The Pearson correlation coefficient measures the linear relationship between adjacent pixel values and defined as in Equation 44:


(44) $$r=\frac{\sum (x_{i}-\mu_{x})(y_{i}-\mu_{y})}{\sqrt{\sum {\left(x_{i}-\mu_{x}\right)}^{2}\sum {\left(y_{i}-\mu_{y}\right)}^{2}}}$$

Ideally, *r* ≈ 0 indicates no correlation. In our experiment, *r =*0.005, confirming that the encrypted image lacks linear pixel dependencies, which enhances its resistance to statistical attacks.

### 6.5 Detection performance evaluation

This study evaluated the YOLOv11n-12D model using **Tables 3**, **4**, confirming its innovative breakthroughs. **Table 3** highlights the model’s superior performance in detecting four eggplant diseases: rot (mAP@0.5=0.861), fruit borer (0.872), healthy plants (0.911), and notably thrips (0.753), a 6.5% improvement over the baseline. It achieves accuracy comparable to YOLOv12s (gap <2%) via knowledge distillation while remaining lightweight. **Table 4** provides a comprehensive performance comparison. The model maintains near-teacher accuracy (1.2% mAP@0.5 difference) and achieves a 2.7ms inference speed—3.6× faster than YOLOv12s (9.6ms) and 3.2× faster than YOLOv8n (8.7ms). Its F1-Score (0.804) outperforms YOLOv10n (0.764) and YOLOv8n (0.785), with a 4.5% improvement in the stricter mAP@0.5:0.95 metric, demonstrating stability in multi-scale detection.

**Table 3:** Performance comparison of YOLO models for eggplant disease detection.

Model	Category	Dataset		Performance metrics			
		Images	Instances	Precision	Recall	mAP@0.5	mAP@0.5:0.95
YOLOv11n-12D	Eggplant Rot	117	544	0.838	0.814	**0.861**	0.565
	Eggplant Fruit	237	323	**0.861**	0.819	**0.872**	0.407
	Healthy	216	331	0.871	0.886	**0.911**	0.725
	Eggplant Thrips	175	318	0.752	0.643	**0.753**	0.442
YOLOv11n-12D Unencrypted (Baseline)	Eggplant Rot	117	544	0.838	0.814	**0.861**	0.565
	Eggplant Fruit	237	323	0.861	0.819	**0.872**	0.407
	Healthy	216	331	0.871	0.886	**0.911**	0.725
	Eggplant Thrips	175	318	0.752	0.643	**0.753**	0.442
yolov11n	Eggplant Rot	117	544	0.838	0.724	0.822	0.552
	Eggplant Fruit	237	323	0.826	0.807	0.850	0.395
	Healthy	216	331	0.861	0.891	**0.924**	0.728
	Eggplant Thrips	175	318	0.810	0.575	0.707	0.411
yolov12s	Eggplant Rot	117	544	0.852	0.827	0.873	0.582
	Eggplant Fruit	237	323	0.879	0.831	0.884	0.418
	Healthy	216	331	**0.883**	0.901	0.922	**0.741**
	Eggplant Thrips	175	318	0.765	0.659	0.767	0.456
yolov10n	Eggplant Rot	117	544	0.805	0.706	0.781	0.502
	Eggplant Fruit	237	323	0.821	0.768	0.826	0.384
	Healthy	216	331	0.852	**0.904**	0.910	0.719
	Eggplant Thrips	175	318	0.770	0.505	0.672	0.397
yolov8n	Eggplant Rot	117	544	0.820	0.750	0.817	0.524
	Eggplant Fruit	237	323	0.859	0.794	0.857	0.408
	Healthy	216	331	0.815	**0.907**	0.917	0.717
	Eggplant Thrips	175	318	0.781	0.566	0.697	0.399

Bold values are representative or key results.

**Table 4:** Comprehensive performance comparison.

Model version	Precision	Recall	mAP@0.5	mAP@0.5:0.95	F1-score	Inference speed (ms)
YOLOv11n-12D	0.831	0.791	0.849	0.535	0.804	**2**.**7**
YOLOv11n	0.834	0.749	0.826	0.522	0.789	3.3
YOLOv12s	**0.845**	**0.804**	**0.861**	**0.549**	**0.812**	9.6
YOLOv10n	0.812	0.721	0.797	0.501	0.764	3.1
YOLOv8n	0.819	0.754	0.822	0.512	0.785	8.7

Bold values are representative or key results.

Comprehensive analysis of data from both tables demonstrates that YOLOv11n-12D, through the synergistic optimization of knowledge distillation and Focal Loss, successfully overcomes the traditional trade-off between accuracy and efficiency, achieving the innovative breakthrough of “teacher-level accuracy with edge-level efficiency” and providing reliable technical support for real-time disease detection in smart agriculture.

### 6.6 Information entropy analysis

The formula for information entropy as defined in Equation 45:


(45) $$\text{H}(X)=-\sum_{i=0}^{\mathrm{255}}p(x_{i})\log_{2}p(x_{i})\;\;(Information\;entropy\;(unit:\;bit))$$

The entropy of the original image ranges from 5 to 7, while the encrypted image’s entropy is close to 8 (theoretical maximum), showing a more uniform and random pixel distribution. Our calculated entropy value is 7.6195, indicating high information entropy, which helps prevent information leakage and statistical analysis attacks.

## 7 Conclusion

This paper proposes an integrated system for eggplant disease detection that combines image encryption and deep learning-based recognition. The system employs a lightweight encryption scheme based on 3D chaotic mapping and pixel permutation to secure image transmission with low computational overhead. It then utilizes an optimized YOLOv11n-12D model to process the decrypted images, achieving high detection accuracy and real-time performance. A teacher–student knowledge distillation strategy is incorporated to further enhance model robustness. Experimental results demonstrate that the system not only safeguards data privacy but also outperforms existing methods in accuracy, speed, and stability, offering a reliable solution for smart agriculture. At the same time,our future research will focus on enabling disease detection directly on encrypted images to eliminate the risk of data leakage during decryption. This will involve exploring privacy-preserving techniques like homomorphic encryption and designing lightweight models that can operate effectively in the encrypted domain.

## 8 Discussion

Our study proposes a framework that integrates image encryption with deep learning based object detection for real time, privacy-preserving crop disease monitoring. The designed 3D chaotic cube encryption scheme demonstrates strong security performance, achieving high entropy (7.6195), low pixel correlation (0.0084), and strong resistance to statistical and differential attacks (NPCR = 99.63%, UACI = 32.85%). Meanwhile, the YOLOv11n-12D model retains the detection performance of the teacher model while achieving fast inference speed (2.7 ms), with a notable mAP improvement of +6.5% in small-object detection such as eggplant thrips. This solution offers a promising approach for advancing smart agriculture in rural or resource limited areas. By encrypting images before transmission and decrypting them only during model inference, the framework strikes a practical balance between data security and operational efficiency. Its compatibility with edge devices further supports deployment in real world scenarios, where data privacy, bandwidth limitations, and low computing resources are common challenges. Despite the promising results, the current framework still requires decryption before detection, which introduces a temporary risk of data exposure. Future work will focus on privacy preserving deep learning techniques that support inference directly in the encrypted domain, such as homomorphic encryption or secure multi party computation. Further validation on larger and more diverse crop datasets is also needed to assess generalization. Enhancing the interpretability of both the detection model and the encryption process will help improve transparency and user trust in practical applications.

## Data Availability

The original contributions presented in the study are included in the article/supplementary material. Further inquiries can be directed to the corresponding author.
